# The Onset of Enhanced Intestinal Permeability and Food Sensitivity Triggered by Medication Used in Dental Procedures: A Case Report

**DOI:** 10.1155/2012/265052

**Published:** 2012-09-12

**Authors:** Aristo Vojdani, Jama Lambert

**Affiliations:** ^1^Immunosciences Lab., Inc., 822 S. Robertson Boulevard, Suite 312, Los Angeles, CA 90035, USA; ^2^Cyrex Labs., LLC., 5040 N. 15th Avenue, Suite 107, Phoenix, AZ 85015, USA

## Abstract

Enhanced intestinal permeability and food sensitivity are two of the many proven causes of gastrointestinal disorders. This present report describes a woman with no previous gastrointestinal (GI) complaints, who underwent dental root canal, bone graft, and implant procedures. Postsurgery she experienced an allergic reaction to the combined medications. In the weeks that followed, she presented with multiple food intolerances. Four weeks after the final dental procedure, she was assessed serologically for mucosal immune function, salivary, and blood-gluten reactivity, intestinal permeability, and other food sensitivities. Compared to her test reports from two months prior to her first dental procedure, the patient's results showed high total secretory IgA (SIgA) and elevated salivary antibodies to alpha-gliadin, indicating abnormal mucosal immunity and loss of tolerance to gluten. Her serologic assessments revealed immunoglobulin G (IgG) and IgA antibodies to a range of wheat/gluten proteins and peptides, gut bacterial endotoxins and tight junction proteins. These test results indicate gut dysbiosis, enhanced intestinal permeability, systemic gluten-reactivity, and immune response to other dietary macromolecules. The present case suggests that patients who experience severe allergic or pseudoallergic reactions to medication should be assessed and monitored for gut dysfunction. If left untreated this could lead to autoimmune reactions to self tissues.

## 1. Introduction

Adverse reaction to many antibiotics and local analgesics have been reported in some patients [1–6]. Haptens are chemicals with very small molecular weights. By themselves, haptens do not elicit immune responses; however, they may do so by binding to other larger molecules such as tissue antigens or food proteins [7]. There is potential for interaction between haptens and food proteins with respect to the induction of oral tolerance by regulatory T cells and associated cytokines. It is also postulated that haptens can impact protein tolerance mechanisms through their ability to bind to soluble proteins or peptides within the gut-associated lymphoid tissue [7].

Although rare [1], anesthesic reactions are a growing concern [8]. Adverse reactions have been categorized as follows: toxic (drug overdose, rapid absorption, and intravascular injection), psychogenic, idiosyncratic, and allergic [2]. The usual trigger of the reactions is the paraben or sulfite preservatives in the anesthetics [1]. Reactions may range from psychomotor responses to Type I, Type II, Type III, and Type IV allergic reactions [1–3]. 

These responses can occur at the first dose, but they occur more commonly after subsequent doses, indicating that these drug-specific IgE antibodies require a period of sensitization, whereas some cytopenias are caused by IgG or IgM antibody immune responses [6], which manifest days or weeks after dosing. Females have been shown to be more reactive than men, which may be due to a possible role played by estrogen, or that perhaps the use of cosmetics exposes women to certain chemicals that prime their immune systems to respond (cross-react) to structurally similar chemicals in anesthetics [8]. Here we report a case in which adverse reaction to local analgesics and antibiotics resulted in increased intestinal permeability, gluten sensitivity, and immune reactivity to different proteins and peptides.

## 2. Case Presentation

A 52-year-old woman was given her annual wellness visit by her internist. Based on the medical examination and a normal CBC, chemistry (including liver enzymes), and an autoimmune profile, she was classified as a healthy person. Gluten antibodies were not measured at that time. A few months later she went to her dentist for a root canal, bone graft, and preparation for dental implantation. During five different visits over ten days she was treated with anesthetic material (mepivacaine), antibiotics and painkillers. Four months later the dental implant procedure was completed using local anesthesia with lidocaine and a subsequent prescription of antibiotic (amoxicillin) and painkillers. Four hours following the procedure, she developed a severe allergic reaction with localized edema, in particular the lips, and periorbital area swelling. The patient became agitated and exhibited a generalized itching, particularly her face, hands and feet. Tightness of the chest with wheezing and difficulty in breathing was an indication of allergic reaction to one or more of the medicines used. She was immediately treated with 0.01 mL per kg of body weight of adrenaline, intramuscularly supplemented by antihistamine treatment. However, while the allergic reaction was controlled, the patient developed severe vomiting and diarrhea with intense abdominal pain, which lasted for 8 days. Two weeks later, while the diarrhea had ameliorated, the patient continued to complain about bloating and abdominal discomfort with irritable bowel-like syndrome. She was referred to a GI specialist who detected nothing of note after a thorough physical examination, CBC, and blood chemistry. The possibility of gluten-sensitivity was then considered, and the patient was tested for HLA typing and IgG and IgA antigliadin and antitransglutaminase (anti-tTG) antibodies. Basic immunological tests showed: IgG antigliadin 6.8 U/mL (normal range < 20 U/mL); IgA anti-gliadin 4.1 U/mL (normal range < 20 U/mL); IgG anti-tTG 2.1 U/mL (normal range < 6 U/mL); IgA anti-tTG 1.2 U/mL (normal range < 4 U/mL); negative for HLA DQ2 and DQ8. Based on these findings gluten-sensitivity and celiac disease were excluded, and it was concluded that the patient was suffering from psychogenic or idiosyncratic reaction associated with reaction to the anesthetic and its synergistic effect with the antibiotics. The patient was still complaining about bloating and abdominal pain, particularly 1–3 hours after each meal, and thus she sought a second opinion. A standard test was ordered for ASCA and p-ANCA IgG, which are the suggested tests for suspected Crohn's disease and ulcerative colitis; and comprehensive tests from Cyrex Labs were ordered, including occludin/zonulin IgG, IgM, IgA, actomyosin IgA and endotoxin or lipopolysaccharides IgG, IgM, IgA, which indicate intestinal barrier damage and gut dysbiosis, a comprehensive assessment of gluten-reactivity, and a panel of IgA + IgG against 24 foods. While ASCA and p-ANCA were completely within the normal range, results from Cyrex testing using enzyme-linked immunosorbent assay (ELISA) IgG- and IgA-antibody testing against an array of wheat and associated antigens showed IgG positivity against 8 out of 12 tested antigens and IgA against 6 out of 12 tested antigens at 2–7 fold higher than established reference ranges (see Table 1). Additional Cyrex testing resulted in indications of mucosal immune upregulation, gut dysbiosis, enhanced intestinal permeability, and multiple systemic immune reactivity to dietary macromolecules (see Table 2). Frozen oral fluid and serum specimens collected during the previous visit were sent to Cyrex Labs for analysis to determine the patient's preoperative baselines for comparison with the postoperative results (see Table 2). Based on elevation in antibodies, a clinical nutritionist advised the patient to take probiotics and go on a restricted diet free of glutens, wheat-germ agglutinin (WGA), and other foods. Six months after the introduction of the diet and the probiotics, retests were ordered (see Table 2); the patient's GI discomfort had subsided, and she was back to normal health.

## 3. Discussion

This case report differs from other documented reactions to antibiotics and analgesics in that we describe the resulting increased intestinal permeability and the onset of gluten-sensitivity and immune reactivity to various dietary macromolecules. 

Several case reports on patient reactions to the use of multiple drugs have been published [2, 4–6]. A proposed mechanism for these reactions is cross-reactivity [4, 5] with other drugs or foods. There is documentation of patients exhibiting adverse reactions to many chemically unrelated antibiotics [6]; however, the label of multiple drug allergy syndrome remains controversial and not fully documented [4, 6]. Whether reaction is from inflammatory immune responses or physical/psychological stress brought on by the procedure [8, 9], both mechanisms have been shown to play a role in the mucosal dysfunction that leads to increased intestinal permeability. For the patient in this case report, the synergistic effect of multiple hapten exposure and stress contributed to abnormal mucosal immune function and the breakdown in immunological tolerance to dietary proteins and peptides.

The term gluten sensitivity refers to a state of heightened immunological responsiveness to gluten as indicated by the elevation of IgG, IgA, or both against gliadin but not against transglutaminase [10]. Gluten sensitivity begins with the loss of mucosal immune tolerance to wheat antigens and peptides due to environmental factors affecting the mucosal immune homeostasis. In the present case gluten sensitivity was confirmed based on GI symptoms and immunological testing (see Table 1). The pre and postoperative results for this present case show that the synergistic effects of anesthetics, antibiotics, and painkillers appear to have resulted in dysregulation of her mucosal immune system, followed by a breakdown in immunological tolerance to wheat and other dietary proteins and peptides. This, possibly in combination with the effect of environmental factors on the activity of the digestive enzymes, resulted in the induction of the opening of tight junctions and the entry of undigested wheat proteins and peptides into the submucosa, lymph nodes, and the circulation. These antigens were subsequently presented by antigen-presenting cells to T cells and B cells. During this process, gliadin-specific B cells are assisted by gliadin-specific T cells, leading to B-cell clonal expansion and the release of IgG and IgA antibodies to gliadin and associated proteins and peptides, which in this case was detected about four weeks after the traumatic event.

We conclude that screening for enhanced intestinal permeability to macromolecules in patients with GI discomfort associated with the use of anesthetics and antibiotics may be performed by measuring circulating antibodies against tight junction proteins, LPS, and actomyosin. The consequence of enhanced intestinal permeability is increased immune reactivity to multiple dietary antigens resulting in antibody production against a variety of foods. If this condition is left undetected, the consequence of haptens binding to foods and human tissues, and the entry of these into circulation can trigger autoimmune response. 

## Figures and Tables

**Table 1:** Results from an array of wheat and enzyme antigens.

Antigen	IgG reactivity	IgA reactivity
Wheat	+	+
Wheat germ agglutinin	+	+
*α*-gliadin 33 Mer	−	−
*α*-gliadin 17 Mer	+	−
*γ*-glidain 15 Mer	+	+
*ω*-gliadin	−	−
Glutenin	+	+
Gluteomorphin	+	+
Prodynorphin	+	+
Transglutaminase	−	−
Gliadin-tTG complex	−	−
Glutamic acid decarboxylase 65	+	−

**Table 2:** Pre- and postoperative results for mucosal immunity, intestinal permeability, and food sensitivity as expressed in ELISA units.

Lab measurements	Before	4 weeks after dental procedure	6 months after probiotics, digestive enzymes	Normal range in ELISA units
Secretory IgA	32	117	46	16–48
Gliadin IgA + IgM	21	129	33	7–18
Transglutaminase IgA + IgM	16	23	17	7–18
Occludin/zonulin IgG	37	92	35	6–33
Occludin/zonulin IgM	31	86	28	6–47
Occludin/zonulin IgA	26	135	67	6–31
Lipopolysaccharide IgG	41	116	45	3–48
Lipopolysaccharide IgM	39	73	43	3–49
Lipopolysaccharide IgA	46	143	87	6–34
Actomyosin IgG	8	6	9	<20
24 foods IgA + IgG	++	+++++*	++	+

*Multiple dairy antigens, hemp, gluten-containing grains, millet, amaranth, quinoa, yeast, tapioca, oats, coffee, rice, and potato.
